# Cognitive performance in aged rats is associated with differences in distinctive neuronal populations in the ventral tegmental area and altered synaptic plasticity in the hippocampus

**DOI:** 10.3389/fnagi.2024.1357347

**Published:** 2024-02-26

**Authors:** Claudia Sagheddu, Tamara Stojanovic, Shima Kouhnavardi, Artem Savchenko, Ahmed M. Hussein, Marco Pistis, Francisco J. Monje, Roberto Plasenzotti, Mohammed Aufy, Christian R. Studenik, Jana Lubec, Gert Lubec

**Affiliations:** ^1^Division of Neuroscience and Clinical Pharmacology, Department of Biomedical Sciences, University of Cagliari, Monserrato, Italy; ^2^Programme for Proteomics, Paracelsus Medical University, Salzburg, Austria; ^3^Division of Pharmacology and Toxicology, Department of Pharmaceutical Sciences, University of Vienna, Vienna, Austria; ^4^Institute of Pharmacology, Pavlov First Saint Petersburg State Medical University, St. Petersburg, Russia; ^5^Department of Zoology, Faculty of Science, Al-Azhar University, Asyut, Egypt; ^6^Section of Cagliari, Neuroscience Institute National Research Council of Italy (CNR), Cagliari, Italy; ^7^Unit of Clinical Pharmacology, University Hospital, Cagliari, Italy; ^8^Center for Physiology and Pharmacology, Department of Neurophysiology and Neuropharmacology, Medical University of Vienna, Vienna, Austria; ^9^Division of Biomedical Research, Medical University of Vienna, Vienna, Austria

**Keywords:** dopamine, LTP, learning and memory, *in vivo* electrophysiology, VTA GABAergic and VTA glutamatergic neurons, dementia

## Abstract

**Introduction:**

Deterioration of cognitive functions is commonly associated with aging, although there is wide variation in the onset and manifestation. Albeit heterogeneity in age-related cognitive decline has been studied at the cellular and molecular level, there is poor evidence for electrophysiological correlates. The aim of the current study was to address the electrophysiological basis of heterogeneity of cognitive functions in cognitively Inferior and Superior old (19-20 months) rats in the ventral tegmental area (VTA) and the hippocampus, having Young (12 weeks) rats as a control. The midbrain VTA operates as a hub amidst affective and cognitive facets, processing sensory inputs related to motivated behaviours and hippocampal memory. Increasing evidence shows direct dopaminergic and non-dopaminergic input from the VTA to the hippocampus.

**Methods:**

Aged Superior and Inferior male rats were selected from a cohort of 88 animals based on their performance in a spatial learning and memory task. Using *in vivo* single-cell recording in the VTA, we examined the electrical activity of different neuronal populations (putative dopaminergic, glutamatergic and GABAergic neurons). In the same animals, basal synaptic transmission and synaptic plasticity were examined in hippocampal slices.

**Results:**

Electrophysiological recordings from the VTA and hippocampus showed alterations associated with aging *per se*, together with differences specifically linked to the cognitive status of aged animals. In particular, the bursting activity of dopamine neurons was lower, while the firing frequency of glutamatergic neurons was higher in VTA of Inferior old rats. The response to high-frequency stimulation in hippocampal slices also discriminated between Superior and Inferior aged animals.

**Discussion:**

This study provides new insight into electrophysiological information underlying compromised cerebral ageing. Further understanding of brain senescence, possibly related to neurocognitive decline, will help develop new strategies towards the preservation of a high quality of life.

## Introduction

In the last decades, improved medical care and lifestyle contributed to extending life expectancy (Friedman, 2020). The process of aging is highly variable and not easily predictable, being influenced by several factors, ranging from genetics to lifestyle and dietary habits and incidental events. Preservation of well-being, independence, and high quality of life relies on the maintenance of molecular and functional processes underlying affective and cognitive brain activity (Cabeza et al., 2018; Aron et al., 2022).

Among other brain regions, the limbic system has been shown to be compromised throughout the lifespan (Penner and Mizumori, 2012; Bettio et al., 2017). The limbic system operates as a hub amidst affective and cognitive facets by processing sensory inputs related to motivated behaviors and memory (McLachlan, 2009). In this regard, the midbrain ventral tegmental area (VTA) is a major source of dopamine projections, either by unique-or by GABA/Glutamate co-release (Sulzer et al., 1998; Tritsch et al., 2012; Cai and Ford, 2018) to subcortical limbic structures, such as the nucleus accumbens and the hippocampus, which are implicated in positive/negative reinforcement and memory, respectively (Scatton et al., 1980; Swanson, 1982; Itoh et al., 1984; Verney et al., 1985; Gasbarri et al., 1994; Lammel et al., 2014; Tsetsenis et al., 2022). Additionally, a subset of non-dopaminergic neurons has been shown to send projections from the VTA to the hippocampus (Ntamati and Luscher, 2016), although their functional relevance remains to be fully elucidated.

Impairment of these brain areas has been associated with age-related pathological conditions, such as dementia and Alzheimer’s disease (AD). Besides the well-known degeneration of the hippocampus (Jaroudi et al., 2017; Josephs et al., 2017; Rao et al., 2022), impairments of the VTA and the dopaminergic system in AD have been proposed (Nobili et al., 2017; Cordella et al., 2018; Bozzali et al., 2019; Krashia et al., 2019; Gloria et al., 2021; La Barbera et al., 2022; Spoleti et al., 2024). Indeed, understanding age-related mechanisms in the VTA and limbic projections could provide novel preventive and/or therapeutic approaches (Cordella et al., 2018; La Barbera et al., 2021; Spoleti et al., 2022), a major goal to counteract age-related cognitive decline as well as dementia and AD (Scheltens et al., 2021). Accordingly, our previous findings indicate that modulation of the dopamine system by the selective dopamine transporter (DAT) inhibition could prove beneficial to restoring several brain alterations associated with aging and age-related cognitive decline (Sagheddu et al., 2020; Lubec et al., 2021; Kouhnavardi et al., 2022; Lubec et al., 2023; Sagheddu et al., 2023).

In this scenario, we took advantage of our validated method to separate superior-and inferior-performing rats (Lubec et al., 2019, 2021) as a model of healthy versus compromised aging. By combining behavioral and *in vivo* as well as *ex vivo* electrophysiological procedures, we investigated the functional outcomes of different cell populations in the VTA and hippocampal plasticity.

## Materials and methods

### Animals

Male Sprague–Dawley rats (88 rats with age of 19–20 months and 10 rats 12 weeks old at the beginning of the hole-board experiment) were bred and maintained in the Core Unit of Biomedical Research, Division of Laboratory Animal Science and Genetics, Medical University of Vienna. Rats were housed in groups of two in standard Makrolon cages filled with autoclaved woodchips (temperature: 22 ± 2°C; humidity: 55 ± 5%; 12 h artificial light/12 h dark cycle: light on at 7:00 am). Aged rats were on a low-calorie diet (ssniff, R/M-H Ered II, Soest, Germany) from fourth month of age. Tap water and food was provided *ad libitum*. All behavioral experiments were performed during the light phase of the light–dark cycle. The study was carried out in accordance with the 2010/63/EU guidelines, evaluated by the ethics committee of the Medical University of Vienna, Austria and approved by the Federal Ministry of Education, Science and Culture, Austria (66.009/0018-V/3b/2019).

### Hole-board test

Animals were habituated to the experimental room at least 24 h prior to the start of the experiment. Food intake was restricted to reduce the weight of the rats to 85% of their initial body weight. A hole-board test was performed as previously described (Lubec et al., 2019) with minor modifications. The hole-board with the dimension of 1 m × 1 m was manufactured of a black plastic board bounded by a translucent plexiglass wall, with proximal spatial cues (black/white symbols) and surrounded by room structures that served as distal cues. A second board below the testing arena was scattered with food pellets to avoid olfactory orientation. Light was adjusted to 15 lux.

The test started with handling (15 min per day for four consecutive days) to acclimatize animals to human interaction. During the following 2 days, animals were habituated to the hole-board apparatus [free exploration of the maze for 15 min each day with access to food pellets (dustless precision pellets, 45 mg, Bioserv®, Flemington, NJ, USA)]. The training was conducted over four consecutive days (five trials on day 1, four trials on day 2, three trials on day 3 and one trial on day 4) with an inter-trial interval of 20 min. Four out of 16 regularly arranged holes (diameter and depth 7 cm) were baited. The pattern of baited holes remained the same during the entire experiment. Each trial lasted 2 min or until all four pellets were found; exception was the first trial on day 1 (lasting 4 min). The board was cleaned with 1% Incidin® after each trial in order to remove any olfactory cues. All trials were videotaped and the number of hole-visits was manually counted. Reference memory index (RMI) was calculated using the formula (first + revisits of baited holes)/total visits of all holes. Animals’ tracks were acquired using the AnyMaze video-tracking system (Stoelting Co., IL, USA).

### Open field

The open field consisted of a black wooden board (1.20 m × 1.20 m) surrounded by white wooden walls (50 cm in height); light was adjusted to 15 lux. Each animal was placed in the center of the open field and behavior was recorded for 10 min. Data analysis was performed using the AnyMaze software (Stoelting Co., IL, USA); total distance traveled, immobility time and numbers of inner zone crossing were compared between the groups.

### Voluntary sucrose intake test

The voluntary sucrose consumption was determined 1 day after conducting the open field. Rats were given a free choice between two bottles for 24 h, one containing tap water and another a 2% sucrose solution. Fluid (sucrose solution or tap water) consumption was calculated by weighing the bottles before and after exposure to the animals. The preference for sucrose was calculated as a percentage of the consumed sucrose solution relative to the total amount of liquid drunk.

### *In vivo* electrophysiological recordings

Electrophysiological recordings were performed 1–3 months after finishing behavioral tests. Rats were anesthetized with chloral hydrate (300 mg/kg, i.p. and additional doses when necessary) and placed in the stereotaxic apparatus (Kopf, Tujunga, CA, USA). The scalp was retracted and one burr hole was drilled above the ventral tegmental area in the midbrain (AP, +2.0 mm from the tip of the lambda, 0.4–0.6 mm from midline; V, 7.5–8.5 mm from cortical surface), according to the stereotaxic rat brain atlas (Paxinos and Watson, 2007). Single unit activity of neurons was extracellularly recorded with glass micropipettes filled with 2% Pontamine sky blue (Merck, Sigma-Aldrich C8679) dissolved in 0.5 M sodium acetate. Individual action potentials were amplified (DAM80 World Precision Instruments, Sarasota, FL, USA and A-M Systems 1700) and displayed on a digital storage oscilloscope (HM507, Hameg Instruments). Experiments were sampled online and offline with Spike2 software (Cambridge Electronic Design, Cambridge, UK) by a computer connected to a CED 1401 interface (Cambridge Electronic Design, Cambridge, UK). To estimate cell population spontaneous activity, the electrode was passed in 6–9 predetermined tracks separated by 200 μm and the number of active cells encountered in each track was averaged. At the end of the recording session, direct current was passed through the recording micropipette to mark the position of the electrode.

#### Isolation of putative dopamine neurons

Single putative dopamine cells were identified according to previously published criteria, such as broad action potential with triphasic positive–negative–positive deflections and duration from the start to the negative trough between 1.6 and 2.4 ms (Ungless et al., 2004). The pattern of activity, with firing rate < 10 Hz, was regular, irregular or bursty. Bursts were estimated as the occurrence of two spikes at interspike interval < 80 ms and terminated at interspike interval > 160 ms (Grace and Bunney, 1984a,b). Response to aversive stimuli was tested by applying a 2 s nociceptive mechanical pinch to the hind paw contralateral to the recording brain hemisphere.

#### Isolation of putative glutamatergic neurons

Single putative glutamatergic neurons of the VTA were identified as biphasic with positive–negative deflections and duration from the start to the negative trough between 0.8 and 1.2 ms (Ungless et al., 2004). The pattern of activity, with relatively high spontaneous firing rate < 25 Hz, was regular or irregular (Luo et al., 2008).

#### Isolation of putative GABAergic interneurons

Putative GABA-containing neurons were identified according to typical biphasic negative–positive deflections and relatively regular spontaneous firing rate < 10 Hz (Steffensen et al., 1998; Luo et al., 2008).

### *Ex vivo* electrophysiological recordings

Immediately after finishing *in vivo* recording, animals were decapitated, and brains were extracted and placed into an ice-cold artificial cerebrospinal fluid (aCSF) solution composed of (mM): 125 NaCl, 2.5 KCl, 20 NaHCO_3_, 2.5 CaCl_2_, 1 MgCl_2_, 25 D-glucose, 1 NaH_2_PO_4_ (pH 7.4). Hippocampi were dissected and slices of 400 μm thickness were sectioned perpendicular to the long axis of the hippocampus using the McIlwain tissue chopper (TC752, Campden Instruments LTD., Loughborough, England). Slices were transferred to customized recovery chamber, submerged in aCSF and the chamber was placed in a water bath set at 30°C. Slices were allowed to recover for at least 1 h before electrophysiological measurements. The aCSF solution was continuously bubbled with a carbogenated gas mixture (95% medical O_2_ + 5% medical CO_2_). The recordings were done in a small submersion chamber continuously perfused at 3–4 mL/min with aCSF (30 ± 2°C). Only slices from dorsal hippocampus were used for recordings.

Evoked field excitatory post-synaptic potentials (fEPSPs) were recorded in the *stratum radiatum* of the hippocampal CA1 area via aCSF-filled glass micropipettes (3 ± 1 MΩ) made of capillary-glass (Harvard Apparatus GmbH, Hugo Sachs Elektronik, Germany) in a horizontal puller (P-87, Sutter Instrument, Novato, CA). Electrical stimulation was delivered via a customized Teflon-coated tungsten wire bipolar electrode (⁓50 μm tip diameter) to the CA3 Schaffer collateral axons. Electrical stimulus was generated by an ISO-STIM 01D stimulator (NPI Electronics, Tamm, Germany). Electrophysiological measurements were obtained using an AxoClamp-2B amplifier and digitalized using a Digidata-1440 interface (both from Axon Instruments, Molecular Devices, Berkshire, UK). Data was acquired and analyzed using the pClamp-11 software (Molecular Devices, Berkshire, UK).

Input/output (I/O) curves were generated by plotting the fEPSPs slope values against the increasing square pulses (200 μs, 15 s inter-pulse interval, 0–9 V and 1 V increments) of electrical stimulation. Input values eliciting 40–50% of the maximum field-slope response were used to examine short-term synaptic plasticity, basal synaptic transmission and to deliver high-frequency stimulation to induce potentiation.

To study hippocampal short-term synaptic plasticity, paired-pulse facilitation (PPF) field responses were examined. To this aim, two electrical stimulation pulses with increasing inter-pulse intervals of 20, 40, 60, 80, and 100 ms were delivered.

The protocol used to induce long-term potentiation (LTP) consisted of 5 bursts of electrical stimulation [each with 10 electrical pulses delivered at 100 Hz (200 μs/pulse)] presented at intervals of 500 ms.

LTP was assessed by examining the temporal evolution of the fEPSP measurements before and after LTP induction. For this, the fEPSP values obtained after LTP-induction were normalized to the average of the fEPSP values obtained before LTP-induction (baseline). Changes in the fEPSP slopes (initial decaying phase) were used for quantitative analyses. fEPSPs measurements for baseline, PPF and those obtained after LTP induction were acquired at a frequency of 0.033 Hz. fEPSP slope values across slices (3 to 5) were averaged to yield a single data point for each animal. Two-way ANOVA with Tukey’s post-hoc test was used to identify differences between experimental groups.

### Statistical analysis

No statistical method was used to predetermine sample size. The sample size was chosen based on previous studies in our laboratory. Old animals were not randomly allocated to experimental groups but based on the criteria described in the results section. All experiments were performed by experimenters who were not aware of the assignment to the experimental groups. Data were tested for normal distribution by Shapiro–Wilk test. Differences between means were considered significant at the *p* < 0.05. All data are expressed as mean ± SEM and were analysed using GraphPad Prism 7 software (GraphPad Software, CA, USA). Detailed statistical parameters for specific experiments are described in the appropriate sections or figure legends.

## Results

### Cognitive status of aged and young rats

**Figure 1:**
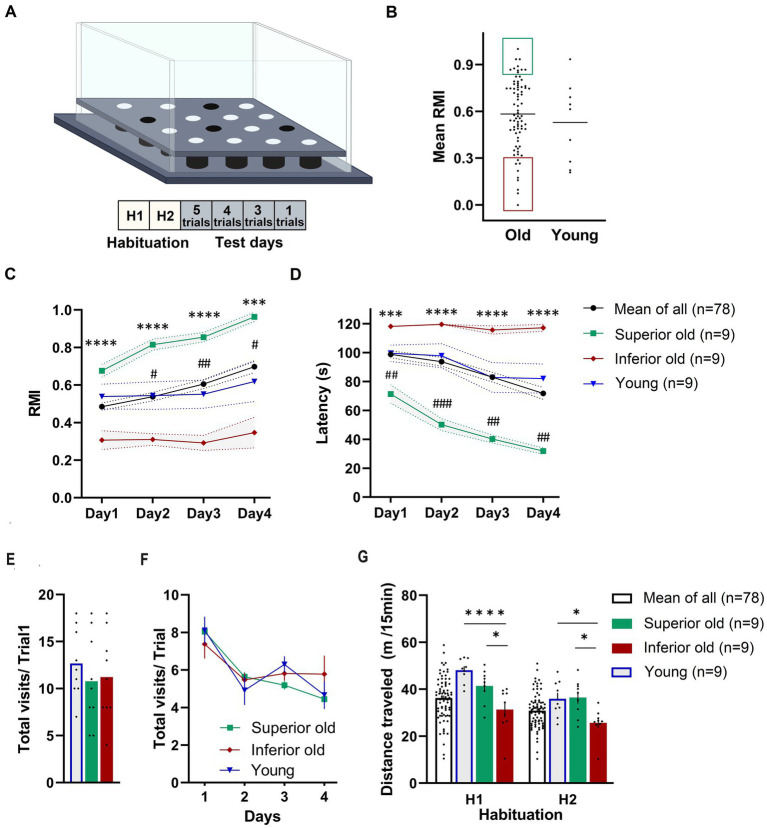
**(A)** Schematic demonstration of hole-board maze. Animals have to learn and remember the position of four baited holes during four test days and 13 trials. **(B)** From a cohort of 88 male Sprague–Dawley rats (19–20 months old) “Superior” (green area) and “Inferior” (red area) performers were selected based on their mean reference memory index (RMI) derived from trial 6, 10, and 13 ± 1 standard deviation. Ten aged rats were excluded from the analysis because they did not fulfill the criteria. Individual points represent specific animals and the line indicates the mean of mean RMIs. Performance of Young, Old (Mean of All), Superior old and Inferior old rats in the hole-board test as expressed by RMIs **(C)** and latency **(D)** to find all pellets. Two-way RM-ANOVA followed by Tukey’s post-hoc tests, # Superior old vs. Young, * Superior old vs. Inferior old. Total numbers of hole dips in the first trial **(E)** and over four days of training **(F)**. **(G)** Food-deprived animals were habituated to hole-board in two sessions (H1 and H2; one session per day) and distance traveled was recorded. Two-way ANOVA followed by Sidak’s multiple comparison test. Values are expressed as mean ± SEM; *n* = 9 per group; ^#/^*p < 0.05, ^##^*p* < 0.01, ^###/^***p < 0.001, ****p < 0.0001.

The effect of aging on spatial learning and memory in 19–20-months-old male Sprague–Dawley rats was evaluated in the hole-board, a food-motivated spatial learning and memory task. The apparatus and the training protocol are presented in Figure 1A. A group of 88 aged rats was trained in a hole-board, and their performance was scored based on their mean reference memory indices (RMI) derived from trials 6, 10 and 13 of the retrieval phase (trials following 24 h after previous training session) (Figure 1B). Old rats having mean RMI one standard deviation above the mean were characterized as Superior (*n* = 9), whereas rats having mean RMI one standard deviation below the mean were characterized as Inferior performers (*n* = 9). “Intermediate” performers were used for treatment with novel dopamine transporter inhibitors (not published). Rats with less than 52 hole entries in total over the 13 trials were excluded from the analysis. Based on this criterium, one animal from the young group and 10 from the aged rats were removed from the analysis. Performance of Young rats (*n* = 9) was analyzed without separation into groups. No significant differences were detected when comparing the mean RMIs of Young and Old rats (Mann–Whitney test, *p* > 0.05; Figure 1B).

Figures 1C,D presents the behavioral data in terms of RMI and latency to complete the task. A two-way ANOVA for repeated measures (RM) on RMI and latency revealed a significant overall difference between four groups [*F*_(3,101)_ = 12.72, *p* < 0.0001; *F*_(3,101)_ = 13.79, *p* < 0.0001; RMI and latency, respectively] and a significant day effect [*F*_(2.084,210.5)_ = 10.57, *p* = 0.0001; *F*_(2.292,231.5)_ = 15.98, *p* < 0.0001; RMI and latency, respectively]. Superior rats performed significantly better than Inferior (Tukey’s *post-hoc* test, *p* < 0.0001; RMIs and latency) ones. The group of Young rats and Old rats (a whole group of 78 aged rats) performed significantly worse than Superior old rats (*p* < 0.05) over all 4 days of the training. No significant differences were detected between Young and Old rats (*p* > 0.05 over all 4 days). A total number of hole dips in the trial 1 (only trial unaffected by the learning process; Figure 1E) and over 4 days of training (Figure 1F) was not significantly different between the groups; therefore, differences in RMIs between the groups cannot be simply attributed to different motivation, however, also cannot be excluded.

We also compared horizontal activity during two habituation sessions (H1 and H2). Both, young and old animals explored the maze significantly more in the first (H1, novelty) session (*p* < 0.05). A two-way RM-ANOVA revealed a significant overall difference between the groups [*F*_(2,24)_ = 9.112, *p* = 0.0011] and a significant “session” effect [*F*_(1,24)_ = 71.86, *p* = 0.0001]. Sidak’s *post-hoc* test revealed significant differences in distance traveled between Superior and Inferior, as well as between Inferior old and Young (*p* < 0.05), but not between Superior old and Young (Figure 1G).

### Spontaneous locomotor activity

**Figure 2:**
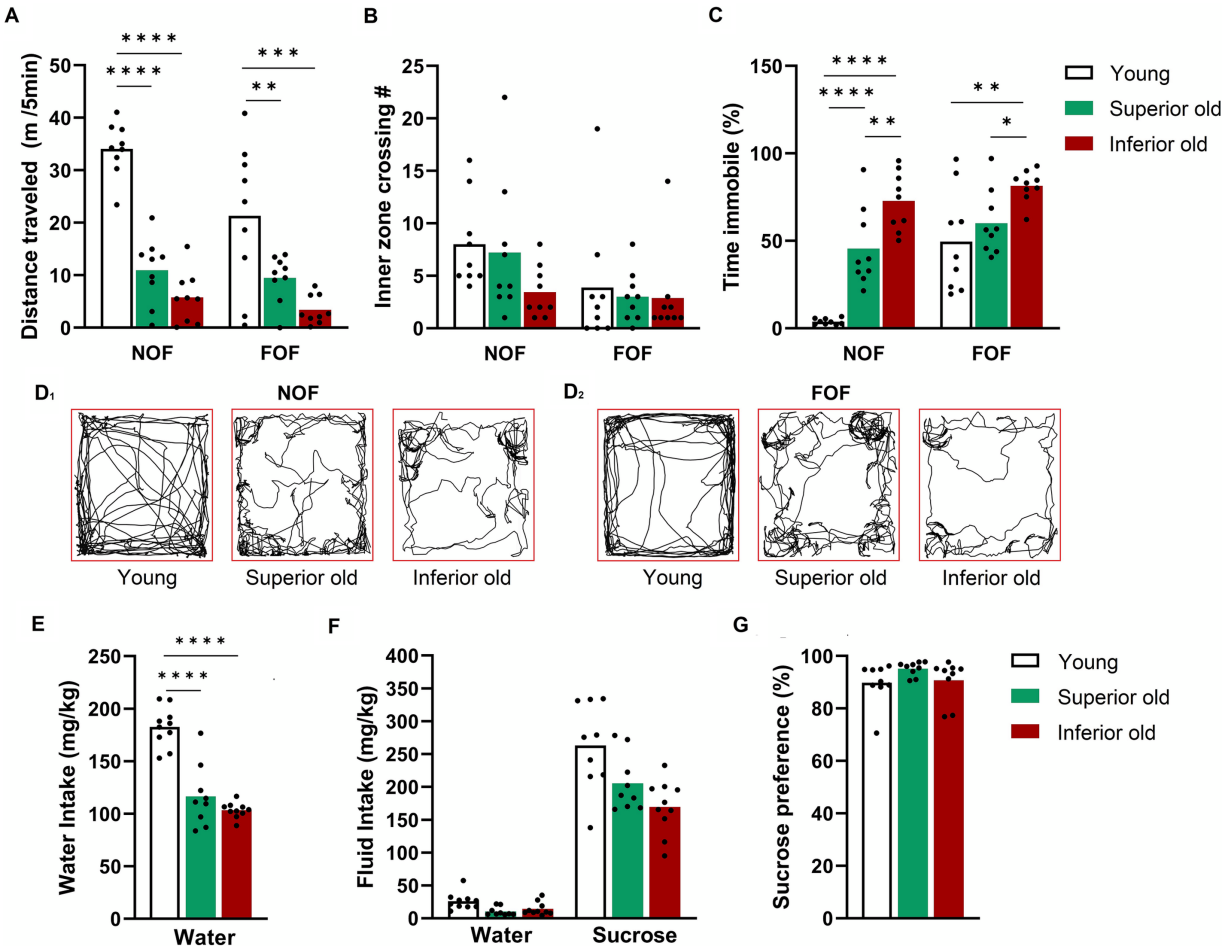
Spontaneous locomotor activity and sucrose-preference test in young and aged male Sprague–Dawley rats. **(A)** Distance travelled, **(B)** number of entries to the inner zone, **(C)** time immobile, and (D1-2) representative traces of locomotor activity recorded in novel open field (NOF) session and familiar open field (FOF) session. Two-way RM-ANOVA followed by Sidak’s post-hoc test for multiple comparisons. **(E)** Water intake (averaged values over three days when only water was provided), **(F)** water and 2% sucrose intake when two bottles presented, **(G)** preference for sucrose. One-way ANOVA with Dunnett’ *post-hoc* test. Data are expressed as a mean with individual data points, *n* = 9 per group, *p < 0.05, **p < 0.01, ***p < 0.001, ****p < 0.0001.

At least 1 week after completing the hole-board test, spontaneous locomotor activity was evaluated in the open field apparatus. Unlike habituation sessions of the hole-board task, animals were not deprived of food, so exploration was not motivated by reward. Animals were not habituated to the apparatus; therefore, we refer to the first test as novel open field (NOF) and to the second test as familiar open field (FOF). There was a major difference in distance traveled among the three groups [two-way RM-ANOVA, *F*_(2,24)_ = 37.53, *p* < 0.0001]. Aged rats showed a decreased amount of locomotor activity as compared to Young (Tukey’s *post-hoc* test, *p* < 0.01); no significant difference between Superior and Inferior groups could be detected (*p* = 0.28 and *p* = 0.18 for NOF and FOF, respectively; Figures 2A–C,D_1_,D_2_). Young rats showed more decrement in locomotion as a function of repeated tests than aged rats (Sidak’s *post-hoc* test, Young *p* = 0.0003, aged *p* > 0.05). There was no significant difference among the groups in number of entries to the inner zone (Figure 2B). Compared to Young, aged rats were less active and spent more time immobile; a significant difference could be detected between Superior and Inferior (*p* = 0.008 and *p* = 0.046 for NOF and FOF, respectively; Figure 2C).

Next, we compared the hedonic phenotype using the two-bottle sucrose preference test, an indicator of anhedonia in rodents. Young rats consumed significantly more water than aged rats (*p* < 0.0001), while water intake did not differ between the two groups of aged rats (Figure 2E). No significant differences in sucrose preference could be detected between the groups (Figures 2F,G).

### Electrophysiological activity of the ventral tegmental area in intact brain

**Figure 3:**
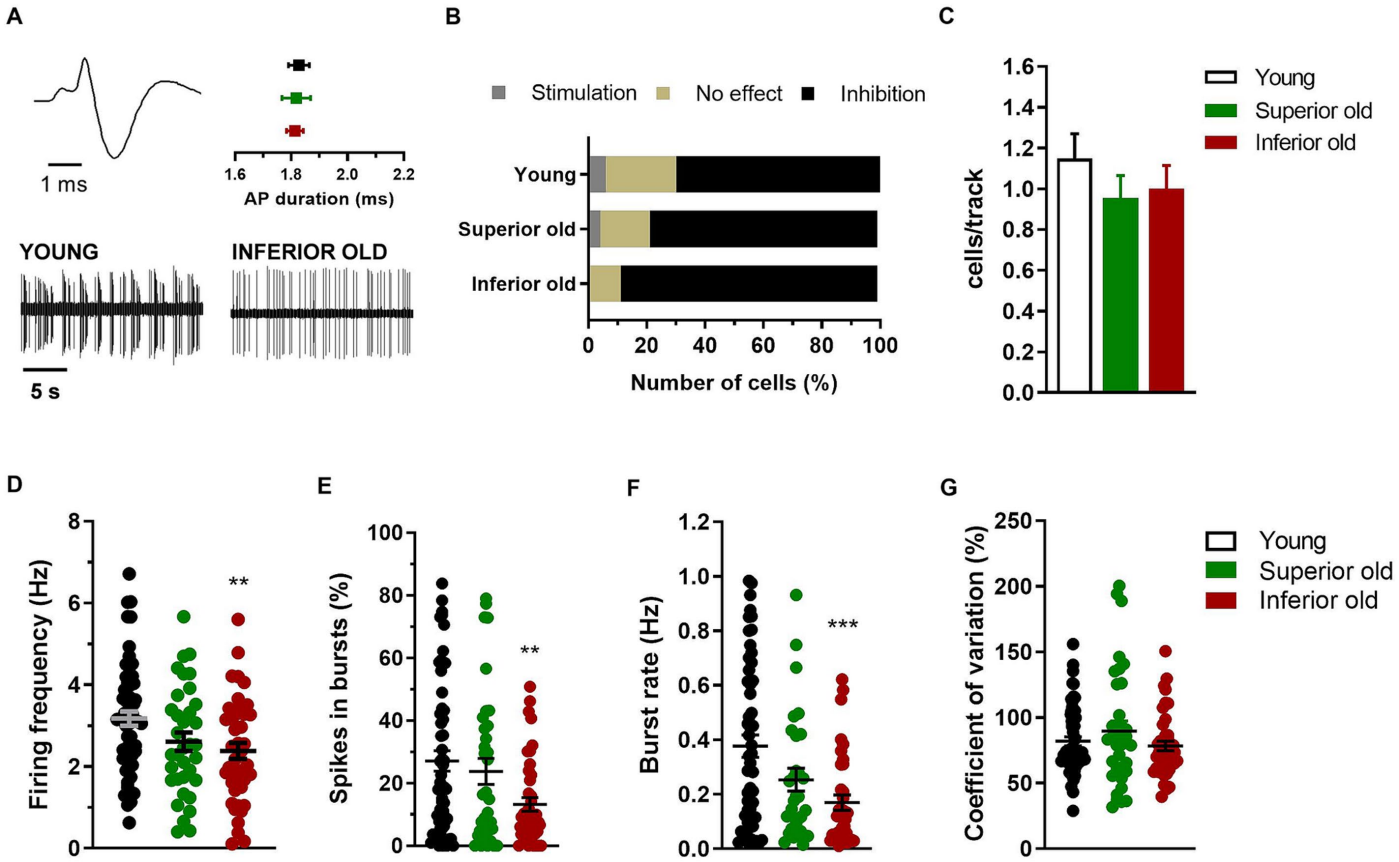
Dopamine neurons in the VTA of young and aged rats. **(A)** Example of typical action potential (AP) waveform (top left) and extracellular recordings (bottom) from different experimental groups (Young *n* = 9; Superior old *n* = 8; Inferior old *n* = 9 rats). The graph (top right) shows the average action potential (AP) duration as measured from the start to the negative trough. **(B)** Percentage of responding/not responding dopamine neurons to the contralateral paw pinch. The *χ*^2^ test on the number of cells (Young *n* = 35 inhibited, *n* = 3 stimulated, *n* = 12 no effect; Superior old *n* = 18 inhibited, *n* = 1 stimulated, *n* = 4 no effect; Inferior old *n* = 30 inhibited, *n* = 0 stimulated, *n* = 4 no effect) showed no difference among groups [χ^2^(4) = 4.49; *p* = 0.34]. **(C)** Bar histograms showing the average number of cells per electrode track [*F*(2, 153) = 0.75, *p* = 0.47]. **(D)** Individual data points and average firing frequency of dopamine neurons [Young *n* = 58; Superior old *n* = 35; Inferior old *n* = 44; *F*(2, 132) = 4.78, *p* = 0.009]. Individual data points and the average **(E)** percentage of spikes in burst [*F*(2, 132) = 5.119, *p* = 0.007], **(F)** burst rate [*F*(2, 117) = 8.003, *p* = 0.0006], and **(G)** coefficient of variation in percentage [*F*(2, 132) = 1.27, *p* = 0.28]. Unless differently stated, data are expressed as mean ± s.e.m. and one-way ANOVA was used to assess differences. Tukey’s multiple comparisons test **p < 0.01, and ***p < 0.001 Inferior old vs. Young.

**Figure 4:**
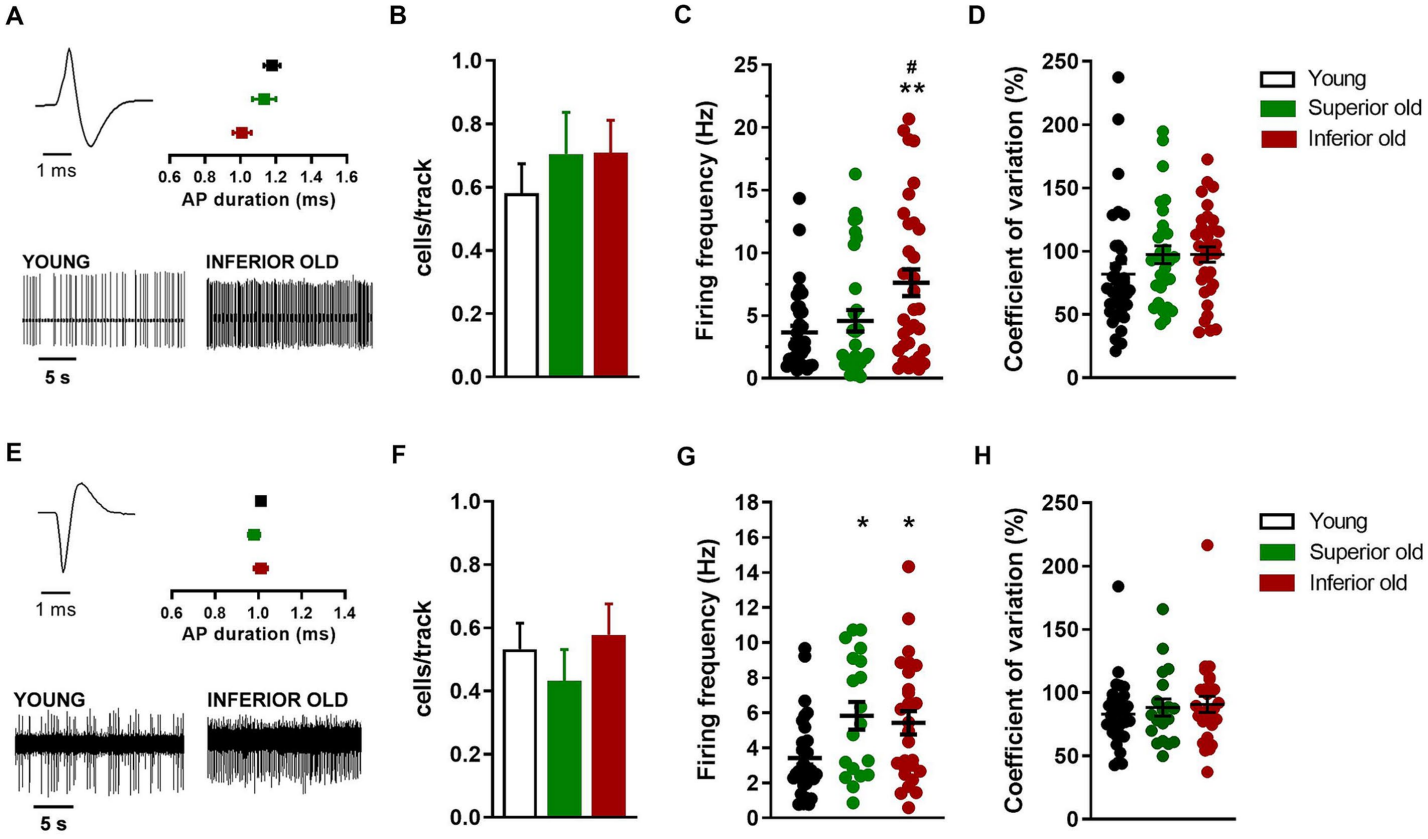
Non-dopaminergic neurons in the VTA of young and aged rats. **(A)** Example of action potential (AP) waveform (top left) and recordings (bottom) from a putative glutamatergic neuron. The graph (top right) shows the average action potential duration as measured from the start to the negative trough. **(B)** Bar histograms showing the average number of glutamatergic neurons per electrode track [*F*(2,151) = 0.49, *p* = 0.61]. **(C)** Individual data points and average firing frequency of glutamatergic neurons [Young *n* = 34; Superior old *n* = 31; Inferior old *n* = 34; *F*(2,96) = 6.09, *p* = 0.003]. **(D)** Individual data points and the average of coefficient of variation in percentage [*F*(2,96) = 1.58, *p* = 0.21]. **(E)** Example of action potential waveform (top left) and recordings (bottom) from putative GABAergic neuron. The graph (top right) shows the average action potential duration as measured from the start to the positive trough. **(F)** Bar histograms showing the average number of GABAergic neurons per electrode track [*F*(2,148) = 0.58, *p* = 0.55]. **(G)** Individual data points and average firing frequency of GABAergic neurons [Young *n* = 35; Superior old *n* = 19; Inferior old *n* = 29; *F*(2,76) = 5.19, *p* = 0.007]. **(H)** Individual data points and the average of coefficient of variation in percentage [*F*(2,76) = 0.53, *p* = 0.58]. Data are expressed as mean ± s.e.m. and one-way ANOVA was used to assess differences; Tukey’s multiple comparisons test *p < 0.05 and **p < 0.01 vs. Young; #p < 0.05 vs. Superior old.

In the same rats (Young *n* = 9, Superior old *n* = 8, Inferior old *n* = 9), the extracellular electrical activity of single cells from the VTA was recorded, and different neuronal populations were sorted out as putative dopaminergic, glutamatergic and GABAergic neurons (Figures 3A, 4A,E, respectively). The response of dopamine neurons to the contralateral paw pinch was not statistically different in the three experimental groups [*χ*^2^_(4)_ = 4.49; *p* = 0.34], being 35 cells inhibited, 3 stimulated, and 12 not responding in Young rats, 18 inhibited, 1 stimulated, and 4 not responding in Superior old and 30 inhibited, 0 stimulated, and 4 not responding in Inferior old (Figure 3B). Similarly, the number of cells per track did not differ [Young *n* = 61, Superior old *n* = 45, Inferior old *n* = 50; one-way ANOVA *F*_(2, 153)_ = 0.75, *p* = 0.47; Figure 3C], suggesting a preserved general activity during aging. The average firing frequency [one way ANOVA *F*_(2, 132)_ = 4.78, *p* = 0.009, Young *n* = 58; Superior old *n* = 35; Inferior old *n* = 44 cells] and the burst firing, expressed both as percentage of spikes in burst [one way ANOVA *F*_(2, 132)_ = 5.119, *p* = 0.007] and as burst rate [one way ANOVA *F*_(2, 117)_ = 8.003, *p* = 0.0006], were lower in the Inferior old group (Figures 3D–F, respectively). The coefficient of variation did not differ [one-way ANOVA *F*_(2, 132)_ = 1.27, *p* = 0.28, Figure 3G].

Concerning the putative glutamatergic neurons in the VTA, the number of cells per track did not differ between among groups [Young *n* = 62, Superior old *n* = 44, Inferior old *n* = 48; one-way ANOVA *F*_(2,151)_ = 0.49, *p* = 0.61; Figure 4B]. The average firing frequency (Young *n* = 38; Superior old *n* = 31; Inferior old *n* = 34 cells) was different [one way ANOVA *F*_(2,96)_ = 6.09, *p* = 0.003; Figure 4C], being higher in the Inferior old group (Tukey’s *post hoc* vs. Young *p* = 0.0033, vs. Superior old *p* = 0.036). However, the pattern of activity, as expressed by the coefficient of variation, was not changed [one-way ANOVA *F*_(2,96)_ = 1.58, *p* = 0.21; Figure 4D].

Finally, the analysis of putative GABAergic neurons in the VTA showed no difference in the number of cells per track [Young *n* = 62; Superior old *n* = 44; Inferior old *n* = 48; one-way ANOVA *F*_(2,148)_ = 0.58, *p* = 0.55; Figure 4F]. The average firing frequency (Young *n* = 35; Superior old *n* = 19; Inferior old *n* = 29 cells) was higher in both Superior and Inferior old as compared with the Young rats [one-way ANOVA *F*_(2,76)_ = 5.19, *p* = 0.007; Figure 4G]. The coefficient of variation was not changed among groups [and *F*_(2,76)_ = 0.53, *p* = 0.58; Figure 4H].

### Differences in basal synaptic transmission, paired-pulse facilitation and LTP between young and aged rats

**Figure 5:**
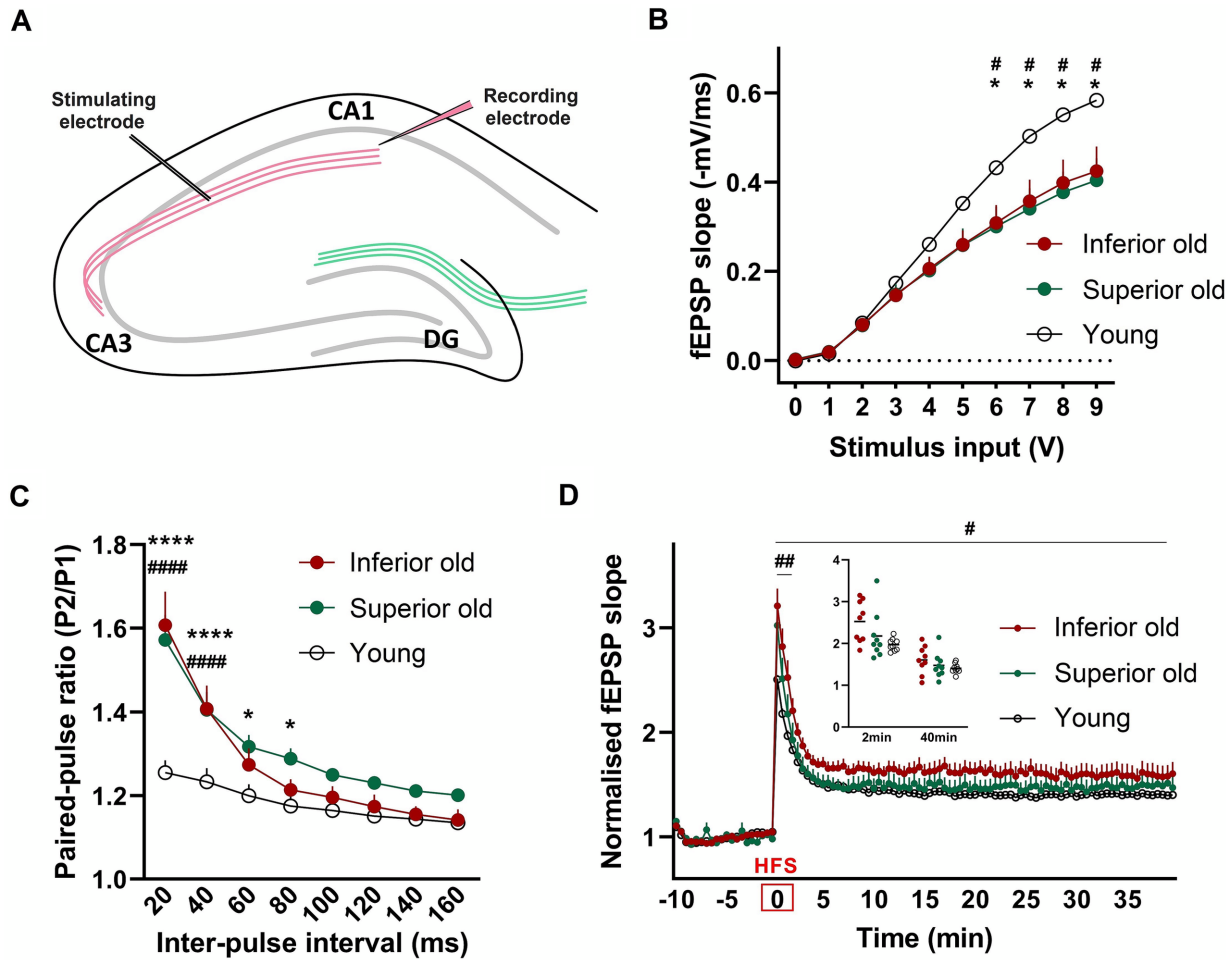
**(A)** For field potential recordings from hippocampal slices, the stimulating electrode was placed in the Schaffer collateral axons of CA3 and a recording electrode in the Stratum radiatum of CA1 **(B)** Input/output (I/O) responses were significantly shifted downward in hippocampal slices from Superior and Inferior performing aged rats. Two-way RM-ANOVAwith Tukey’s post-hoc test. **(C)** Enhanced paired-pulse facilitation in aged animals. Data represent the ratio of the second fEPSP slope to the first fEPSP slope. Two-way ANOVA with Tukey’s *post-hoc* test. **(D)** Potentiation induced after high-frequency stimulation (HFS) was significantly enhanced immediately after HFS (first 2 min) and over 40 min of recording in Inferior old rats compared to Young. Two timepoints (2 min and 40 min) are shown as a dot plot. Two-way RM-ANOVA. * Superior old vs. Young, # Inferior old vs. Young. Data are expressed as a mean ± s.e.m., *n* = 9 per group, ^#/^*p < 0.05, ^##^*p* < 0.01, ^####/^****p < 0.0001.

Basal synaptic transmission and synaptic plasticity were examined by *ex vivo* electrophysiological recordings from hippocampal slices. Field excitatory postsynaptic potentials (fEPSP) were recorded from the dendritic CA1 layer upon stimulation of the CA3-Schaffer collateral pathway (Figure 5A). We observed that the input - output (I/O) curves of fEPSP slopes in response to different stimulation intensities were significantly depressed in two groups of aged rats when compared with those of Young [two-way RM-ANOVA, *F*_(2,24)_ = 3.751, *p* < 0.0382, *n* = 9 per group; Figures 5B,C], suggesting that basal synaptic transmission was impaired in aged rats. However, there was no difference between the two groups of aged animals. To examine possible differences in presynaptic release mechanisms, a paired-pulse stimulation protocol was applied. In aged animals, PPF was significantly enhanced compared to Young [two-way ANOVA *F*_(2,192)_ = 31.03, *p* < 0.0001, *n* = 9 per group; Figure 5C].

To identify changes in synaptic plasticity associated with aging or with the cognitive status of old rats, a high-frequency stimulation (HFS) protocol was applied. The HFS protocol induced robust synaptic strengthening responses in all three experimental groups. We found that post-HFS potentiation was significantly enhanced in Inferior old rats, as compared to Young [two-way RM-ANOVA *F*_(1,16)_ = 5.756, *p* = 0.029; Figure 5D]. No significant differences were observed between Superior old and Young and between the two groups of aged rats. Post-tetanic potentiation (PTP, the increase of the synaptic response that occurs right after high-frequency stimulation) was significantly higher in Inferior old compared to young rats [two-way RM-ANOVA *F*_(1,16)_ = 12.70, *p* = 0.002, *n* = 9 per group].

## Discussion

Spatial learning and memory are among the cognitive functions that decline with age. Although advanced age is commonly associated with intellectual decline, cognitive impairment is not strictly linked to chronological age, and even in a population with minimal differences in genetic variation and environmental factors, as in our case, there is high variability in how the different cognitive functions are preserved or impaired in each individual. In the present study, our established behavioral procedure (Lubec et al., 2019, 2021) was used to identify aged individuals with Superior and Inferior performance in a hole-board task to address the heterogeneity of cognitive aging. Group of young rats was used as a control; however, based on our previous studies (Lubec et al., 2019, 2021; Pretsch et al., 2020), this group showed unusual heterogeneity in the hole-board test that we cannot explain. Therefore, it is questionable whether the group of young rats is representative of the young population in terms of performance in the hole-board task, which provides a strong argument for increasing the sample size in our future studies.

Old Inferior rats with impaired spatial learning and memory showed reduced exploration in both habituation sessions compared to Young and Superior old animals. Reduced exploration in the first habituation session may be related to decreased response to novelty in aged individuals (Rowe et al., 1998). This was supported by results from the open field where old animals tend to spend more time immobile; however, the environmental novelty did not affect the exploration and/or immobility of old rats (as compared to a second OF test). The reduced general activity was not linked to signs of anhedonia, indicating that depression is not associated with age *per se*.

Our experimental protocol for spatial learning and memory involves food deprivation as a stimulus to encourage exploratory behavior. Substantial evidence in animals and humans shows that reward prioritizes events for long-term memory storage (Adcock et al., 2006; Wittmann et al., 2007; Braun et al., 2018). Midbrain dopamine neurons are critically involved in reward processing and have been linked to reward location learning (Martig and Mizumori, 2011). Given that dopaminergic signaling is attenuated with age (Backman et al., 2010) and reduced reward sensitivity has been reported in aged rodents and humans (Eppinger et al., 2011; Gilbert et al., 2011; Tryon et al., 2020), this may to be one of the factors contributing to the behavioral outcome in reward-motivated task.

As midbrain dopamine plays a central role in the regulation of several major biological functions like reward processing, novelty, invigoration of movement and motivation (da Silva et al., 2018) and presumably promotes hippocampal network dynamics associated with memory persistence (McNamara et al., 2014), we examined electrophysiological properties of VTA neurons, including putative dopaminergic, glutamatergic and GABAergic neurons. Many studies suggested that several neuronal subpopulations within the VTA form subcircuits that are differentially involved in specific aspects of behavior and are individually affected in several neuropsychiatric disorders (Bariselli et al., 2016). Results from dopamine neurons in old rats are consistent with previous immunohistochemical and electrophysiological studies, which showed no age-related changes in the number and morphology of TH-positive neurons (Pereira et al., 2021) or in firing parameters (Freeman et al., 1989). However, we evidenced altered electrical properties of dopamine neurons in Inferior aged rats, as expressed by the complete loss of response to an acute nociceptive stimulus, reduced firing activity and bursting activity. This accounts for dopamine system dysfunctions during “unhealthy” aging, possibly associated with cognitive impairments. Reduced dopamine function is consistent with exacerbation of neuropsychiatric symptoms during lifespan, ranging from depressive states to substance use disorders (Skoog, 2011; Alexopoulos, 2019; Tampi et al., 2022). Further, these data are consistent with beneficial electrophysiological and behavioral effects of dopamine enhancement through selective reuptake inhibition in aged rats, including compromised aged rats (Sagheddu et al., 2020; Lubec et al., 2021; Kouhnavardi et al., 2022). To the best of our knowledge, this is the first extensive *in vivo* investigation of non-dopaminergic neurons in the VTA of aged rats. Our data revealed age-related alterations in putative GABAergic neurons as compared with young rats but status-related alterations in glutamatergic neurons in old age. Interestingly, the increased firing frequency of glutamatergic neurons from Inferior aged rats mirrored the reduced firing activity of dopamine neurons in the same area, though correlation is not proven here. The model used here proved robustness to unveil circuit derangements associated with physiological aging and/or brain deterioration, suggesting that the role of non-dopaminergic VTA projections, and particularly their hippocampal branches, deserves further examination. One limitation is that the identification of the different subpopulations of VTA neurons is based solely on the electrophysiological characteristics of the recorded cells.

It is generally assumed that impairment in spatial learning and memory tasks reflects cellular or synaptic dysfunctions in the hippocampus, a region particularly affected by aging. To access age-or cognitive status-related alterations in synaptic functions, we examined evoked field EPSPs at Schaffer collateral CA1 synapses, an area receiving direct dopamine projections from the midbrain (Tsetsenis et al., 2021). There is strong evidence of mechanisms underlying dopaminergic modulation of hippocampal synaptic plasticity (Tsetsenis et al., 2022) and informational flow (Rosen et al., 2015). However, the locus coeruleus (LC) has been described as another source of dopamine in the hippocampus (Kempadoo et al., 2016; Takeuchi et al., 2016; Yamasaki and Takeuchi, 2017; Duszkiewicz et al., 2019), although the LC’s status in the present aging model remains to be addressed.

Here, we show an altered I/O relationship at the CA3-CA1 synapses of aged rats. We assume that the alteration of basal synaptic transmission in aged rats resulted from the decreased probability of neurotransmitter release, as indicated by an increased facilitation index. Notable, Ca2+ is the major trigger of neurotransmitter release and is essential for a variety of other neuronal functions. Aged neurons show a multitude of defects in Ca2+ homeostasis resulting in an increase of Ca2+ loads, which negatively affects neuronal excitability (Moore and Murphy, 2020), increases the threshold frequency for induction of LTP and enhances the vulnerability to the induction of LTD (Nikoletopoulou and Tavernarakis, 2012). In this context, Ca2+ dyshomeostasis has been described as a crucial mechanism underlying cognitive deficits in aging (Uryash et al., 2020). However, changes in basal synaptic transmission and short-term plasticity were specific to advanced age and did not correlate with cognitive status.

Several studies have reported age-related shifts in synaptic plasticity toward reduction of synaptic transmission and decreased ability to induce and maintain LTP (Watson et al., 2002; Burke and Barnes, 2006; Shetty et al., 2017). In addition, reduced LTP after HFS has been associated with impaired spatial memory performance (Bach et al., 1999; Schulz et al., 2002). Controversially, we observed augmented HFS-induced post-tetanic potentiation and LTP in aged compromised rats as compared to Young, while the induction and persistence of LTP in Inferior aged rats were comparable to young animals. However, it should be taken into account that in an effort to examine different brain components in the very same animals, we implemented a methodological approach combining *in vivo* electrophysiological recordings followed by *ex vivo* measurement in acutely dissociated hippocampal slices. Special attention was paid to keeping all the experimental conditions equal across all the studied subjects in order to ensure reproducibility and comparability between the obtained results from the different groups. It is noteworthy to mention that a gradual increase in the degree of cognitive decline and neurodegeneration associated with aging has been analyzed in many different contexts and demonstrated not only in humans but also in various animal models (Kay and Sime, 1962; Ingram et al., 1981; Aggleton et al., 1989; Uttl and Graf, 1993; Chalfonte and Johnson, 1996). Thus, aging itself may influence how different regions of the nervous system respond in different experimental procedures, making it difficult to differentiate up to which extent the observed outputs are due to the physiological nature of the endogenous response at a given age and/or cognitive status or due to how nervous tissue recovers from, e.g., a surgical procedure in an experimental context (Landfield et al., 1978; Deupree et al., 1993; Mori et al., 2000; see also Norris et al., 1996; Kumar and Foster, 2005). The herein-described differences in functional responses observed between experimental groups may thus be due not only to the basal state of the nervous system *per se* but also to how those nervous systems responded to the methodological procedures and pharmacological treatments used, in our case, used anaesthetics. Compounds like choral hydrate (Adrien et al., 1989; LacKamp et al., 2009; Guo et al., 2017; Tamano et al., 2017), isoflurane (Romero-Barragan et al., 2021) or ketamine xylazine cocktails (Alkire et al., 2008; Laverne et al., 2022) are commonly used anaesthetics in electrophysiological studies of brain neuronal circuits. Like several other general anaesthetics, chloral hydrate and its active metabolite trichloroethanol augments GABAergic inhibitory neurotransmission (Peoples and Weight, 1994). Dysregulation of GABAergic signaling in aging is a widely accepted phenomenon, while reduced inhibition in the hippocampus of aged animals (Vela et al., 2003; Stanley and Shetty, 2004) in some instances has been correlated with the cognitive status of aged rats (Haberman et al., 2013; Spiegel et al., 2013). Thus, as mentioned above, a variety of factors could contribute to the results presented here. Further experiments combining several alternative anaesthetic approaches are in demand to expand further the knowledge on the differential effects of different anaesthetics on the properties of synaptic transmission and plasticity across age and brain regions.

Taken together, this study provides novel insights toward the understanding of healthy versus compromised cerebral aging, with a specific focus on the VTA and hippocampus. In this regard, the synergistic use of experimental animal models in tandem with the comprehensive biophysical characterization of the electrical properties of neuronal circuits at brain regions vital for high-order cognitive functions might improve our understanding of the specific impact that aging has on the central nervous system function and its relation to cognitive performance in different population groups.

## Data availability statement

The raw data supporting the conclusions of this article will be made available by the authors, without undue reservation.

## Ethics statement

The animal study was approved by the ethics committee of the Medical University of Vienna, Austria and by the Federal Ministry of Education, Science and Culture, Austria. The study was conducted in accordance with the local legislation and institutional requirements.

## Author contributions

CS: Conceptualization, Writing – original draft, Writing – review & editing, Formal analysis, Investigation, Methodology, Visualization. TS: Investigation, Methodology, Writing – review & editing. SK: Formal analysis, Investigation, Methodology, Writing – review & editing. AS: Investigation, Methodology, Writing – review & editing. AH: Investigation, Methodology, Writing – review & editing. MP: Funding acquisition, Resources, Writing – review & editing. FM: Methodology, Supervision, Writing – review & editing. RP: Project administration, Supervision, Writing – review & editing. MA: Writing – review & editing. CRS: Resources, Supervision, Writing – review & editing. JL: Conceptualization, Formal analysis, Investigation, Methodology, Visualization, Writing – original draft, Writing – review & editing. GL: Conceptualization, Funding acquisition, Project administration, Resources, Writing – review & editing.
